# IL-10 Accelerates Re-Endothelialization and Inhibits Post-Injury Intimal Hyperplasia following Carotid Artery Denudation

**DOI:** 10.1371/journal.pone.0147615

**Published:** 2016-01-25

**Authors:** Suresh K. Verma, Venkata Naga Srikanth Garikipati, Prasanna Krishnamurthy, Mohsin Khan, Tina Thorne, Gangjian Qin, Douglas W. Losordo, Raj Kishore

**Affiliations:** 1 Center for Translational Medicine, Temple University, Philadelphia, Pennsylvania United States of America; 2 Department of Pharmacology, Temple University, Philadelphia, Pennsylvania United States of America; 3 Department of Cardiovascular Science, Houston Methodist Research Institute, Houston, Texas, United States of America; 4 Feinberg School of Medicine, Northwestern University, Chicago, Illinois, United States of America; University of Illinois at Chicago, UNITED STATES

## Abstract

The role of inflammation on atherosclerosis and restenosis is well established. Restenosis is thought to be a complex response to injury, which includes early thrombus formation, acute inflammation and neo-intimal growth. Inflammatory cells are likely contributors in the host response to vascular injury, via cytokines and chemokines secretion, including TNF-alpha (TNF). We have previously shown that IL-10 inhibits TNF and other inflammatory mediators produced in response to cardiovascular injuries. The specific effect of IL-10 on endothelial cell (ECs) biology is not well elucidated. Here we report that in a mouse model of carotid denudation, IL-10 knock-out mice (IL-10KO) displayed significantly delayed Re-endothelialization and enhanced neo-intimal growth compared to their WT counterparts. Exogenous recombinant IL-10 treatment dramatically blunted the neo-intimal thickening while significantly accelerating the recovery of the injured endothelium in WT mice. In vitro, IL-10 inhibited negative effects of TNF on ECs proliferation, ECs cell cycle, ECs-monocyte adhesion and ECs apoptosis. Furthermore, IL-10 treatment attenuated TNF-induced smooth muscle cells proliferation. Our data suggest that IL-10 differentially regulate endothelial and vascular smooth cells proliferation and function and thus inhibits neo-intimal hyperplasia. Thus, these results may provide insights necessary to develop new therapeutic strategies to limit vascular restenosis during percutaneous coronary intervention (PCI) in the clinics.

## Introduction

Defects in endothelial integrity and function are thought to be the initial steps in the pathogenesis of both atherosclerotic lesions and neo-intima (NI) formation after vascular injury. Therefore, strategies to protect the endothelium and/or stimulate its repair after vascular injury have been sought to reduce intimal lesion formation. Several developments have highlighted this effort more excitingly than the advent of percutaneous coronary intervention (PCI), whereby a metallic scaffold device (MESs) alone or sometimes loaded with drug eluting stents (DESs) are deployed to relieve obstructive atherosclerotic lesions, but the effectiveness of PCI is impeded by NI formation, resulting in re-narrowing of the stented artery, a process known as in-stent restenosis (ISR). Although DESs reduce the frequency of ISR, the associated impaired Re-endothelialization (ReEndo) and the resultant risk of stent thrombosis due to the drug and/or the polymer in these devices remain formidable obstacles for complete therapy [1–4].

The role of inflammation in atherosclerosis and restenosis is well established. Mononuclear phagocytic cells are likely participants as a host response during vascular injury, via the secretion of cytokines and chemokines [5, 6]. In this context, TNF-α (hereto referred as TNF), produced largely by activated monocytes/macrophages, is known to be negatively associated with restenosis and atherosclerosis [7–9]. Others and we have previously shown that TNF represses Re-Endo, inhibits endothelial cell (ECs) proliferation and is a strong mitogen for vascular smooth muscle cells (VSMCs) proliferation [10, 11]. Thus exclusive regulation of ECs proliferation and survival within re-vascularized arteries could represent a desirable approach to prevent restenosis.

Interleukin-10 (IL-10), a pleotropic cytokine, strongly deactivates monocytes/macrophages and regulates TNF and other cytokines levels [7, 12, 13]. In mouse model, IL-10 deletion exaggerates inflammatory cytokines accumulation and is associated with a variety of pathogenic outcomes including endotoxemia, intestinal inflammation and atherosclerosis [14–16]. We have previously shown that, IL-10 strongly inhibited inflammatory cells infiltration and TNF expression in denudated mouse carotid arteries [7]. As a molecular mechanism, we demonstrated that IL-10 inhibits HuR expression (a TNF mRNA stablishing protein) and thereby regulates TNF levels [7]. However, the role of IL-10 on ECs proliferation and survival are poorly known and remains to be characterized. Here we report that IL-10 knock out (KO) mice display enhanced injury induced delay in endothelium recovery and resultant neo-intimal thickness. In contrast, systemic IL-10 treatment following carotid artery injury blunts intimal hyperplasia while significantly accelerates ReEndo. Furthermore, at cellular level, IL-10 co-treatment attenuates TNF-induced inhibition of endothelial cell proliferation, cycle arrest, cell death and their binding to monocytes/macrophage while inhibiting TNF-mediated proliferation of vascular smooth muscle cells.

## Materials and Methods

### Animals and carotid artery denudation

Carotid injury was performed in C57BL/6 (WT) or IL-10 KO mice as reported previously [7, 17]. Authors confirm that all animal procedures reported in this study were approved by Temple University Institutional Animal Care and Use Committee (ACUC; protocol approval#4323). All surgeries were performed under appropriate depth of anesthesia, and all efforts were made to minimize suffering. Euthanasia was performed according to approved protocol of carbon dioxide asphyxiation followed by bilateral thoracotomy or cervical dislocation. For IL-10 treatment study, mice were divided into 2 groups and were treated with either 50 μg/kg recombinant murine IL-10 (i.p. injections)/alternate days or with non-immune murine IgG protein as control.

### Histology

To measure the re-endothelialized area, animals were perfused in vivo with Evans blue dye (0.5% Sigma) 5 and 7 days after the injury, as described previously [17]. The areas stained and unstained in blue and the total carotid artery area were measured, and the percentage were calculated using the entire injured area, based on anatomic landmarks, as the baseline. For the measurement of intimal and medial areas, 28 days after the injury carotid arteries were isolated and embedded in paraffin after perfusion fixation with 4% PFA, and sections perpendicular to the long axis of the arteries were cut from the proximal and distal sections of the injured artery. Each section was stained with elastic trichrome (ET) or H&E. Morphometric analysis of digitalized images was performed with the use of NIH ImageJ software.

### Cell culture, antibodies and treatments

U937 human monocyte/macrophage cell line was obtained from ATCC and cultured in RPMI-1640 media supplemented with 10% FBS and antibiotics in 5% CO2 atmosphere. Bovine aortic endothelial cells (ECs) were cultured in MEM medium supplemented with 10% FBS and antibiotics and were treated with recombinant TNF-α (20 ng/mL) with/without recombinant IL-10 (10 ng/mL). For proliferation studies, ECs were rendered quiescent by culturing confluent monolayers in serum free medium for 48–72 h. These synchronized ECs were then re-plated at low densities in medium supplemented with 10% FBS and in the presence or absence of indicated stimuli.

### Thymidine Uptake Assay

Cells proliferation was measured by incorporation of radiolabeled (H^3^) thymidine as described previously [8, 18].

### Constructs, Transient Transfection, and cyclin A Reporter Assays

Cyclin A–Luc promoter reporter constructs containing mouse cyclin A promoter have been described previously [9, 19]. ECs were transiently transfected with luciferase reporter promoter constructs (pGL2-basic) containing mouse cyclin A promoter using lipofectamine (Gibco) following manufacturer’s instructions. Cells were trypsinized, pooled, and re-plated as per experimental requirement and were treated or not with IL-10/TNF-α for 48 hours. Cells were harvested and assayed for luciferase activity using Berthold Lumat LB9501 luminometer. Luciferase activity was normalized to the alkaline phosphatase activity produced by a co-transfected alkaline phosphatase plasmid (pSVAPAP), which served as transfection efficiency control.

### Cell Cycle Analysis

Cell cycle analysis in 48h-starved cells was performed using propidium iodide staining using BD biosciences kit in presence/absence of indicated stimuli.

### TUNEL Assay

TUNEL staining was performed in endothelial cells after IL-10 and TNF treatment as described previously using TUNEL kit from Roche Biotech. [12].

### Statistical Analyses

Results are expressed as the mean ± standard error of the mean (SEM), computed from separate experiments. Comparisons between control and experimental groups were performed using the unpaired t test. Multiple comparisons among treatment groups were performed using one-way analysis of variance (ANOVA) and levels of significance were determined using the Tukey-Kramer multiple comparison post-hoc test if overall comparison across group was statistically significant (GraphPad prism Software Inc., San Diego, CA). P values <0.05 were considered to be statistically significant.

## Results

### IL-10 KO mice display reduced Re-endothelialization and increased neo-intimal hyperplasia in a model of carotid artery injury

**Fig 1:**
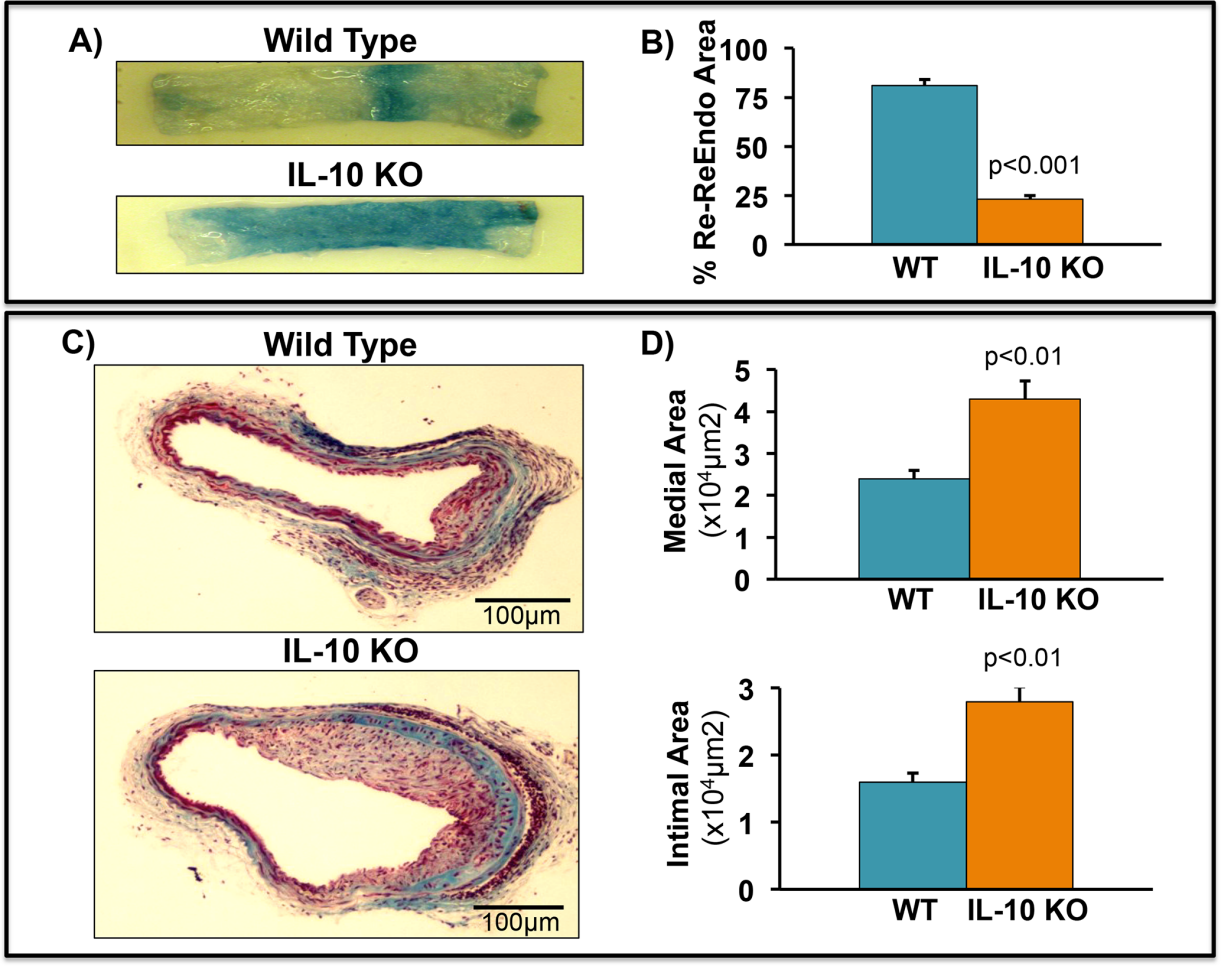
IL-10 deletion reduces ReEndo and increases neo-intimal hyperplasia after carotid artery injury. Carotid injury was performed in WT and IL-10 KO mice by wire injury method. (**A-B)** Mice were injected with Evans blue (0.5%) 2-4h before euthanasia and carotid artery were isolated and visualized for endothelium integrity. Vascular injury and ReEndo was examined on day 7 by Evans blue perfusion method. Enhanced leakage of Evans blue dye from artery in IL-10 KO mice suggested that IL-10 depletion impaired vascular repair. (**C-D)** For morphometric analysis, 28 days after injury carotid artery were isolated and neo-intimal hyperplasia was measured by Elastic Trichome (ET) staining. IL-10 KO mice showed excessive intimal hyperplasia after wire injury. The original magnification of images was 200X. N = 6–8.

We have earlier reported that neutralization of TNF with TNF soluble receptor antibody significantly accelerates ReEndo, inhibits neo-intimal thickening and reduced vascular leakage [10]. Since accelerated recovery of denuded endothelium is required for arterial healing, we examined the role of IL-10 on endothelial recovery in a mouse model of carotid injury. Carotid injury was performed in WT and IL-10 KO mice and ReEndo was examined on day 7 by Evans blue perfusion. IL-10 KO mice showed significant enhanced Evans blue staining after perfusion as compared to WT mice; depicting the reduced endothelial cells growth in IL-10 KO mice seven days post carotid artery injury (**Fig 1A and 1B**). To evaluate the extent of restenosis, we measured the intimal thickening of carotid arteries after injury. Arteries were harvested and elastic trichome (ET) staining was performed to visualized morphological alterations. As shown in representative ET stained sections and quantified in **Fig 1C and 1D**, both intimal and medial areas were significantly larger in IL-10 KO mice compared to WT littermates. These data clearly suggests that IL-10 KO mice showed induced neo-intimal thickening. Our data demonstrates that IL-10 negatively regulates arterial injury-induced restenosis.

### Systemic recombinant IL-10 accelerates endothelial recovery and inhibits neo-intimal thickening in a mouse carotid artery injury model

**Fig 2:**
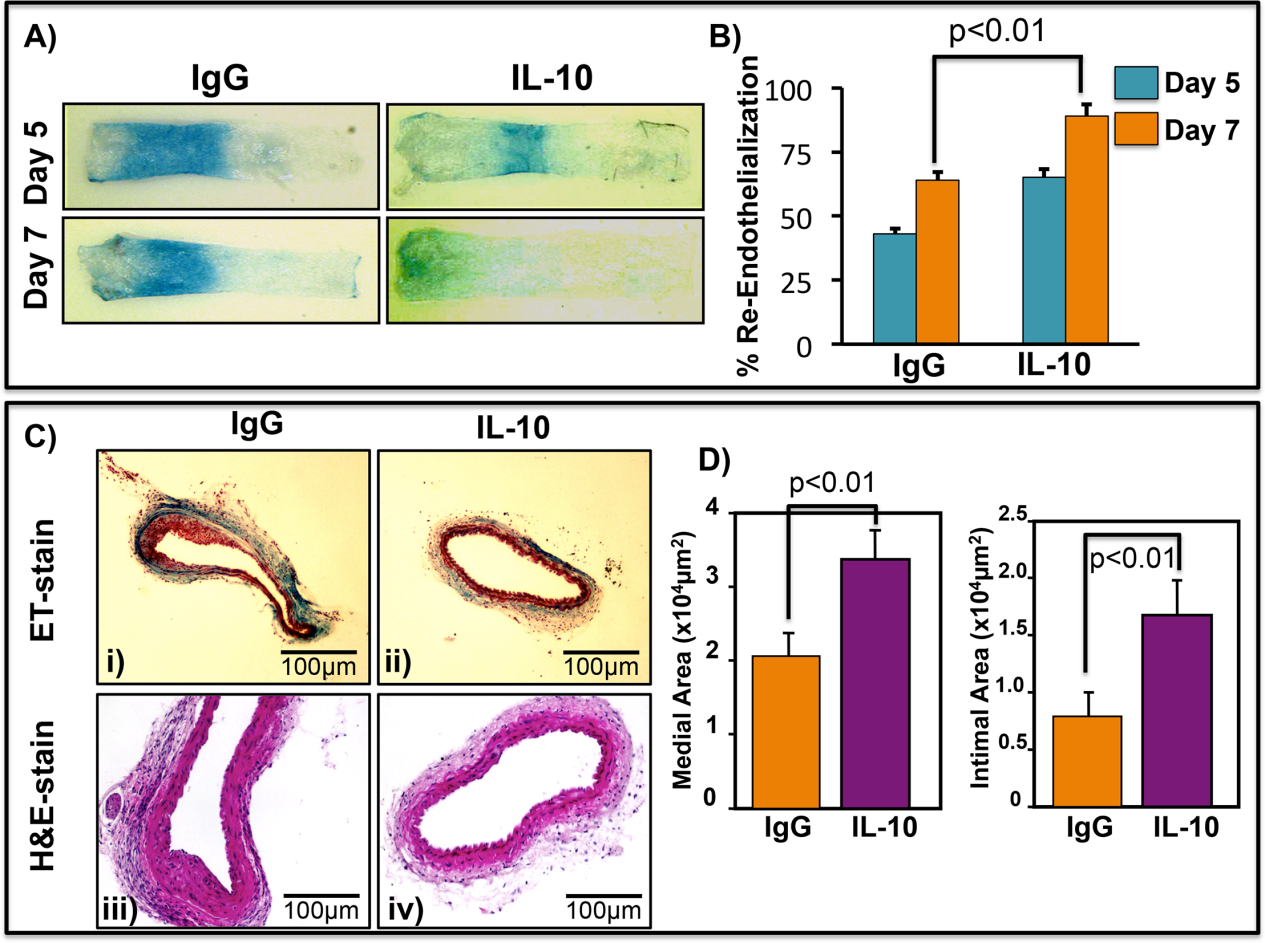
Systemic IL-10 treatment accelerates endothelial recovery and inhibits neo-intimal hyperplasia. ReEndo and intimal hyperplasia in the denuded mouse carotid arteries was measured after IL-10 or IgG (50μg/kg each) (**A**) Representative photomicrographs of Evans blue stained arteries. (**B**) Quantification of re-endothelialized area. ReEndo was examined on days 5 and 7 by Evans blue perfusion method. IL-10 treatment significantly improved ReEndo at both time-points. (**C)** ET and H&E staining of carotid artery sections on 28 days post-injury visualized neo-intimal thickening. Representative photomicrographs of ET (i, ii) and H&E (iii, iv) staining of arterial sections from mice treated with IgG and IL-10, respectively. (**D)** Quantification of medial and intimal areas 28 days post-injury. Recombinant IL-10 treatment attenuated wire injury-induced hyperplasia. The original magnification of images was 200X. N = 6–8.

Furthermore to examine whether systemic IL-10 treatment facilitate endothelium recovery, carotid artery injury was induced in WT mice. Mice were divided into 2 groups and were treated with either 50 μg/kg recombinant murine IL-10 [(i.p. injections), alternate days] or with non-immune murine IgG protein as control. ReEndo was examined on days 5 and 7 by Evans blue perfusion. As shown in **Fig 2A & 2B** ReEndo was significantly faster in IL-10 treated mice compared to control group at each time point (p<0.01), suggesting that IL-10 treatment improves endothelium recovery. Furthermore, intimal and medial thickness was analyzed at day 28 post-injury in mice receiving IL-10 or IgG by elastic trichrome (ET) and H&E staining. As shown in **Fig 2C** (ET stain, panels i and ii; HE stain panels iii and iv) and quantified in **Fig 2D**, IL-10 treatment significantly reduced both medial and intimal areas at day 28, compared to IgG treated mice.

### IL-10 reverses TNF-induced inhibition of endothelial cell proliferation

**Fig 3:**
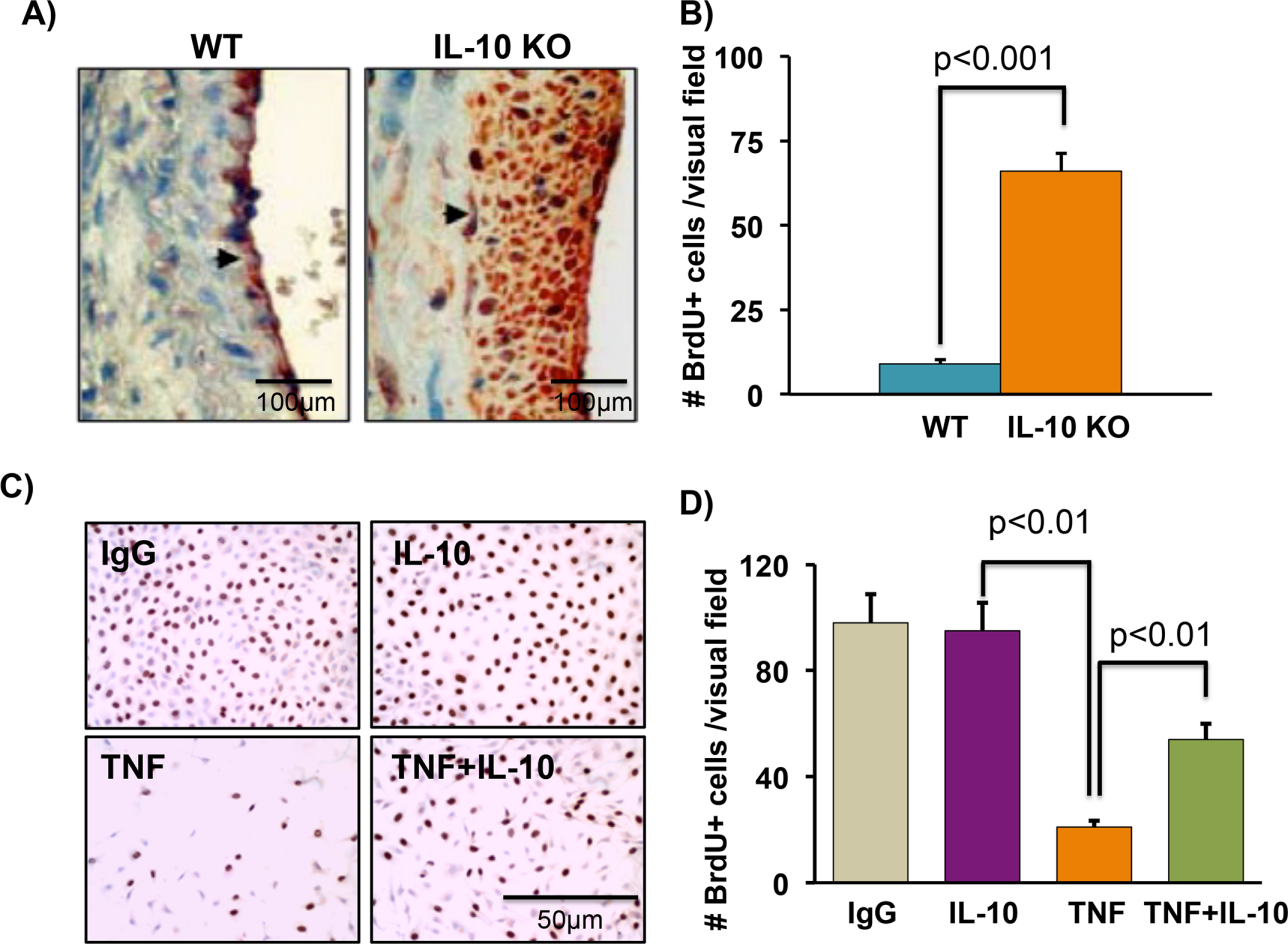
IL-10 treatment improves endothelial cells proliferation after inflammatory insult. Carotid injury was performed in WT and IL-10 KO mice by wire injury method for 28 days. (**A-B)** 24h prior to euthanasia, BrdU (30 mg/kg) was injected in mice intravenously. BrdU positive cells were measured in carotid artery sections using anti-BrdU antibody. IL-10 KO mice showed exaggerated BrdU positive cells. The original magnification of images was 200X. **(C-D)** ECs were rendered quiescent by culturing in the absence of serum for 48h and then stimulated with TNF (20 ng/ml) with or without IL-10 (10 ng/ml) for 24h. Cell proliferation was measured by the incorporation of BrdU. TNF treated ECs showed reduced BrdU positive cells. IL-10 induced ECs proliferation as shown by enhanced BrdU incorporation. The original magnification of images was 200X. N = 4–5.

At cellular level ECs proliferation plays significant role in regulation of restenosis; therefore next we measured the cellular proliferation in WT and IL-10 KO mice after vascular injury. To evaluate cells proliferation, all mice were injected with BrdU (30 mg/kg, Amersham Biosciences) via tail vein 24 h before euthanasia. Arterial sections were immune-stained with anti-BrdU antibodies. IL-10 KO mice showed increased BrdU positive cells after injury (**Fig 3A & 3B**), suggesting that IL-10 loss enhanced intimal cell proliferation. This excessive cellular proliferation could be due to exaggerated growth of smooth muscle cells. To further conform the role of IL-10 on ECs and VSMCs proliferation individually, next we performed sets of in vitro experiments.

**Fig 4:**
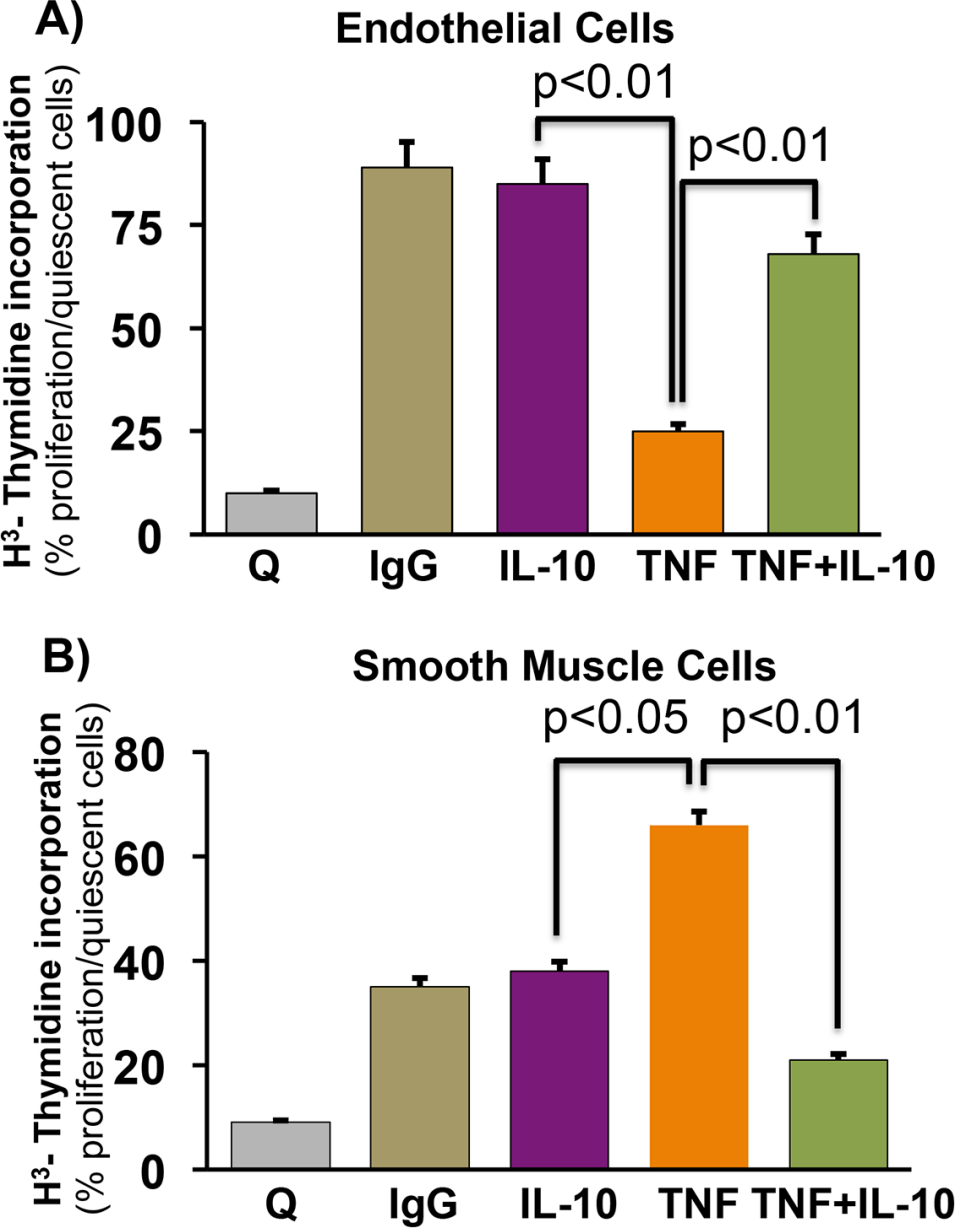
IL-10 differentially regulates endothelial cells and vascular smooth muscle cells proliferation. Endothelial cells and rat vascular smooth muscle cells were rendered quiescent (Q) by culturing in the absence of serum for 48h and then stimulated with TNF (20 ng/ml) with or without IL-10 (10 ng/ml) for 24h. Cell proliferation was measured by the incorporation of H^3^ labeled thymidine. As cells proliferate the radiolabelled thymidine was incorporated in their DNA. (**A)** IL-10 treatment attenuated TNF-induced inhibition of ECs proliferation. (**B)** TNF-induced VSMCs proliferation was inhibited by IL-10. Abbreviations: Q-quiescent, IgG-immunoglobulin G, IL-10-Interleukin 10, TNF-Tumor necrotic factor α. N = 4–5.

We have previously shown that restenosis leads to increased expression of TNF at wound site. Additionally, we have shown that TNF negatively regulates endothelial cell proliferation however; it acts as mitogen for VSMCs but the role of IL-10 on proliferation of these cells type have not been studied [8, 9, 18]. We therefore investigated whether IL-10 treatment reverses TNF-mediated endothelial cell proliferation and improve their functions. ECs were rendered quiescent by culturing in the absence of serum for 48h and then stimulated with TNF with or without IL-10. Cell proliferation was measured by BrdU staining and incorporation of H^3^- thymidine. TNF treatment significantly inhibited ECs proliferation as shown by reduced BrdU immuno-staining; however, this effect of TNF was markedly attenuated by IL-10 treatment **(Fig 3C and 3D).** Furthermore, incorporation of radiolabelled thymidine in proliferating cells was quantified. Cells were treated with TNF and IL-10 alone or in combination in presence of 5 uCi/ml H^3^ thymidine. As shown in (**Fig 4A)**, TNF treatment strongly inhibited radiolabelled thymidine incorporation. Interestingly, IL-10 treatment significantly enhanced thymidine uptake indicating that IL-10 attenuated TNF-induced endothelial cells proliferation (**Fig 4A**). Moreover, since proliferation of smooth muscle cells at the site of injury enhanced the process of restenosis and TNF acts as mitogen for these cell type, next we measured the role of IL-10 on proliferation of rat smooth muscles cells by H^3^ thymidine incorporation assay. As expected, TNF strongly induced smooth muscle cells proliferation and IL-10 treatment inhibited TNF-induced smooth muscle cells proliferation (**Fig 4B**). Our results strongly suggest that IL-10 treatment induced ECs proliferation and inhibited smooth muscle cells proliferation.

### IL-10 rescued endothelial cells from TNF-induced cell cycle arrest

**Fig 5:**
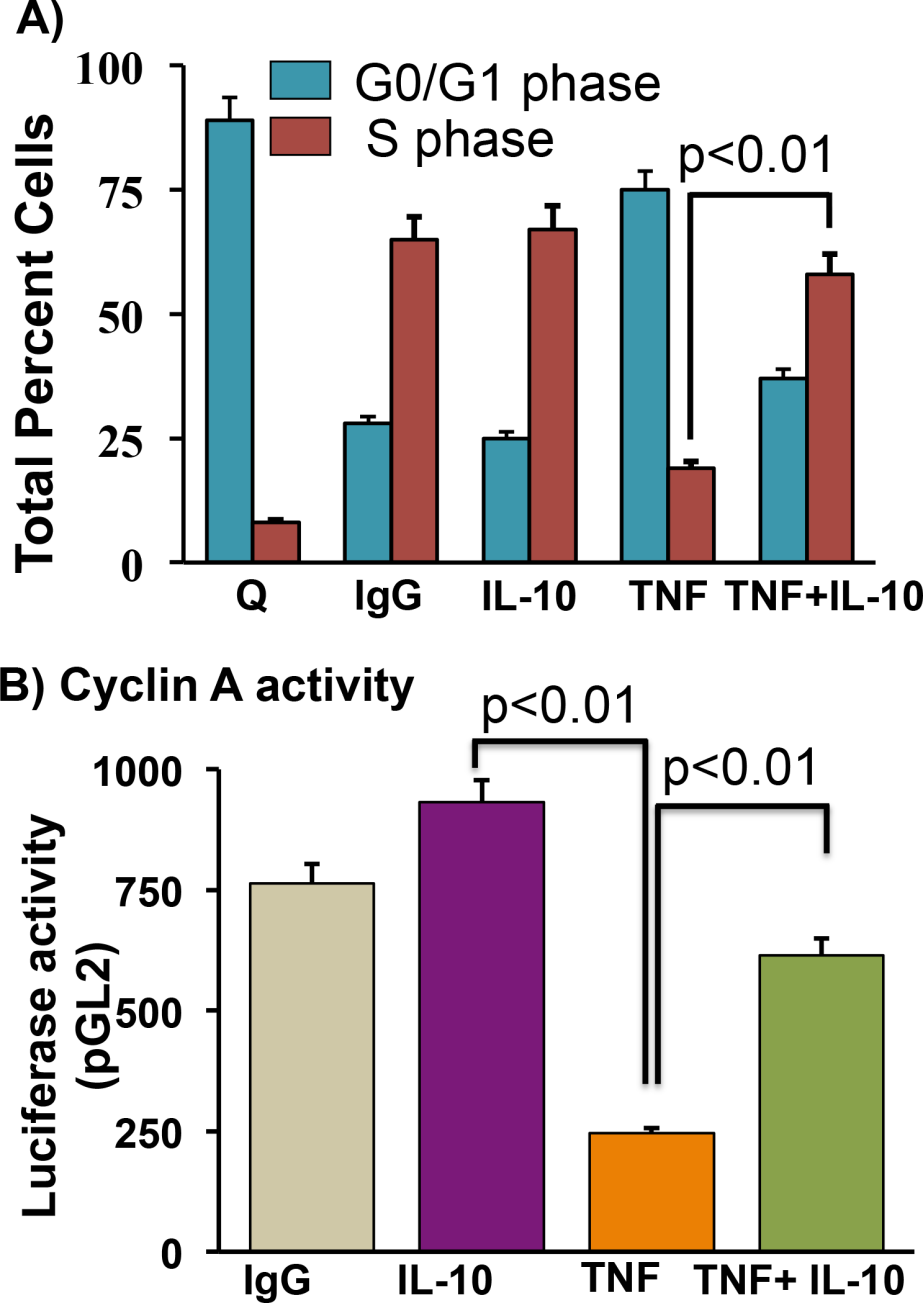
IL-10 rescued TNF-induced endothelial cells cell cycle arrest. ECs were rendered quiescent (Q) by culturing in the absence of serum for 48h and then stimulated with TNF (20 ng/ml) with or without IL-10 (10 ng/ml) for 24 hr. Different stages of cell cycle were measured using BD biosciences cell cycle analysis kit. (**A)** TNF treatment attenuated cell cycle progression as shown by reduced number of cells in S phase. IL-10 treatment attenuated TNF-induced cell cycle arrest as cells are moved from G0/G1 to S phase. (**B)** Cyclin-A activity was measured in ECs by transient transfection with luciferase reporter promoter constructs contain mouse cyclin A promoter. IL-10 treatment attenuates TNF-induced inhibition of cyclin-A activity. N = 4.

Furthermore, we asked whether TNF induces ECs cell cycle arrest. ECs cell cycle analysis was performed using BD biosciences cell cycle analysis kit. TNF treatment strongly inhibited the progress in cell cycle as shown here by increased G0/G1 and reduced S phages of cell cycle. Interestingly, IL-10 co-treatment with TNF reversed TNF-mediated accumulation of EC in G0/G1, with higher cell numbers in S-phase than were observed with TNF-alone treatment, suggesting that TNF-induced cell cycle arrest was attenuated by IL-10 in endothelial cells (**Fig 5A**). Previously others and we have shown that cyclin-A play important roles in regulation of cell cycle progress and TNF markedly inhibited cyclin-A transcriptional activity [9] therefore we further asked whether IL-10 attenuates TNF-induced cyclin A transcriptional activity. ECs were transiently transfected with a cyclin A-promoter luciferase reporter construct (cycAluc-924/+100) as described previously [9] and Cyclin-A activity was measured using luciferase detection method [9]. TNF treatment strongly inhibited cyclin-A transcriptional activity compared to control cells. Interestingly, TNF-induced cyclin-A activity was significantly inhibited by IL-10 treatment (**Fig 5B**).

### TNF-induced endothelial cells death was inhibited by IL-10 treatment

**Fig 6:**
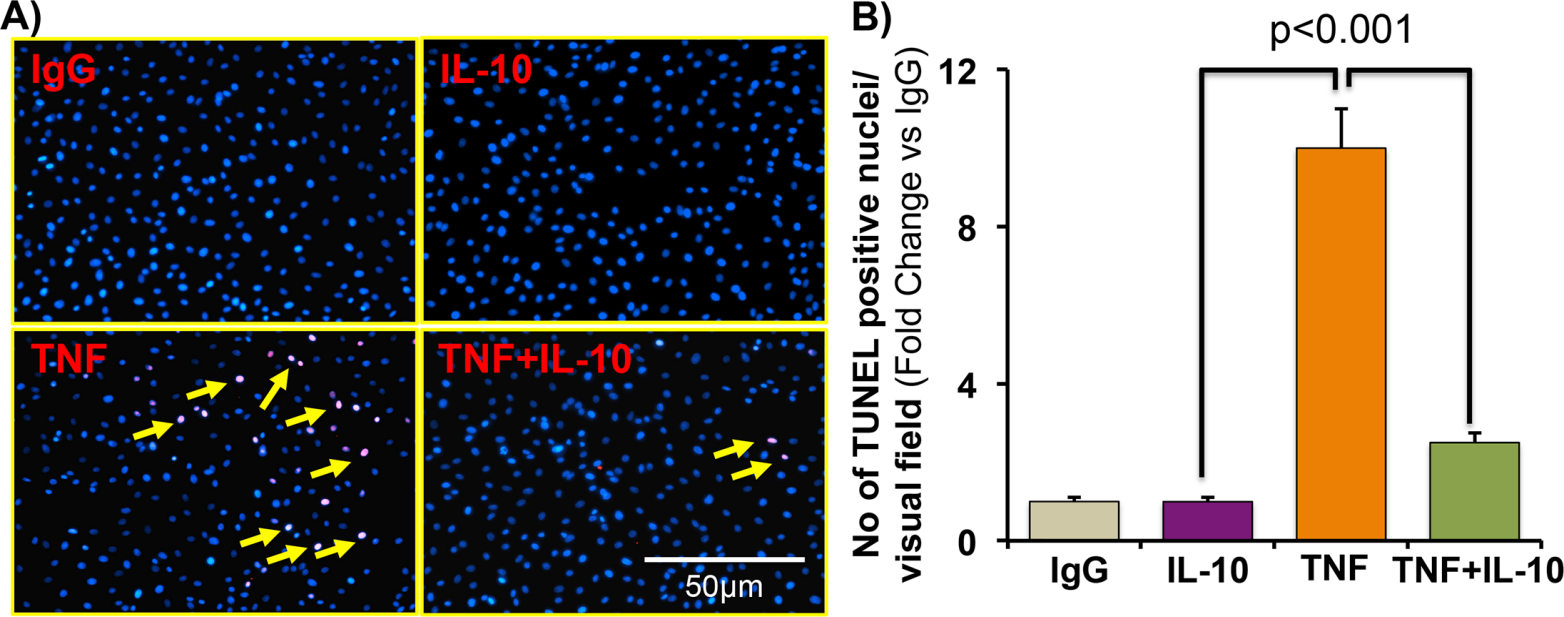
TNF-induced endothelial cell apoptosis is inhibited by IL-10. ECs were stimulated with TNF (20 ng/ml) with or without IL-10 (10 ng/ml) for 24h. After treatments cells were washed with PBS and fixed in 4% formalin for TUNEL staining. ECs death was measured by TUNEL assay kit. **(A-B)** IL-10 attenuates TNF-induced endothelial cells death. Original magnification was 200X. N = 3.

To check whether TNF induces endothelial cells death, we next performed cell death assay in ECs by TUNEL staining after different treatments. TNF treatment strongly activated TUNEL positive cells as compared to control cells. IL-10 treatment strongly inhibited TNF-induced ECs death (**Fig 6A & 6B**).

### IL-10 inhibits TNF-induced monocyte-endothelial cell adhesion

**Fig 7:**
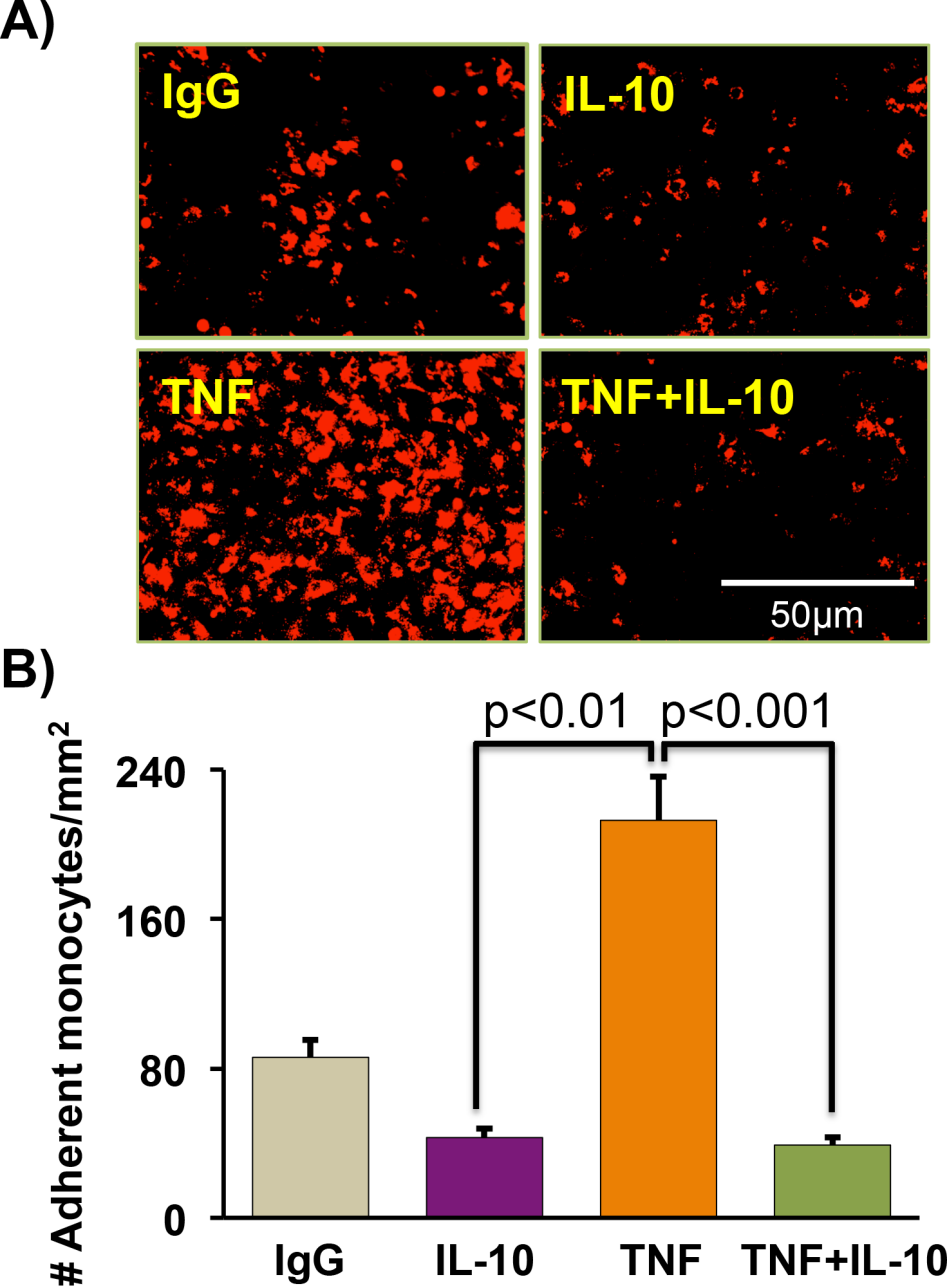
IL-10 reverses TNF-induced monocytes adhesion to endothelial cells. Endothelial Cells (ECs) interaction with monocytes was determined after inflammatory insult. (**A)** ECs were grown to confluency and U937 monocytes, labeled with DiI, were seeded on top of EC monolayers and were cultured for 6 h in the presence of TNF, IL-10 or simultaneously with both cytokines. After treatments, non-adherent cells were washed and culture dishes were visualized under fluorescence microscope to count the adherent monocytes. Original magnification of images was 200X. (**B)** TNF treatment strongly induced the adhesion of monocytes with endothelial cells. IL-10 strongly inhibited the adhesion of monocytes to ECs. N = 4.

Since monocyte adhesion to endothelial cells plays a critical role in the pathogenesis of arteriosclerosis and restenosis, next we tested the effect of IL-10 on the binding of monocytes to the endothelial monolayer. ECs were grown to confluency and U937 monocytes, labeled with DiI, were seeded on top of ECs monolayers and were cultured for 6 h in the presence of TNF with or without IL-10. After treatment period, non-adherent cells were washed and culture dishes were visualized under fluorescence microscope to count the adherent, DiI-labeled monocytes. As shown in (**Fig 7A & 7B)**, TNF treatment induced monocyte binding to ECs as compared to control cells. IL-10 significantly abrogated TNF-induced monocytes binding/interaction with ECs. Taken together, these data demonstrate that IL-10 co-treatment reversed TNF-mediated ECs dysfunction.

## Discussion

Coronary artery diseases (CAD) represent the leading cause of mortality and morbidity in the developed world [20]. Still limited treatment strategies are available to treat this disease. Coronary angioplasty is a procedure used to open the clogged heart arteries by placing stents to help widen the arteries. There are many options and improvements have been made in coronary angioplasty. However, major limitation of angioplasty is in-stent restenosis (ISR), which is characterized by neo-intimal hyperplasia and blood vessel re-narrowing. Additionally, ISR strategies also cause endothelial injury and dysfunction due to lacerations of intimal walls [1, 21] that leads to recruitment and proliferation of inflammatory and smooth muscle cells [22]. In this study we have shown that IL-10 therapy strongly inhibited neo-intimal hyperplasia and improved ReEndo after coronary artery injury. Interestingly, IL-10 differentially regulates proliferation of endothelial and smooth muscle cells. Additionally, IL-10 treatment induced endothelial cells function by increasing cells cycle progression, inducting cyclin-A activity and inhibiting ECs death; all under the influence of TNF stress. Finally, we have shown that IL-10 inhibits TNF-induced adhesion of monocytes/macrophages with endothelial cells.

Inflammatory cells are activated promptly after vascular injury and recruited to the site of injury and release inflammatory mediators such as TNF and other pro-inflammatory cytokines that facilitate VSMCs migration and proliferation and inhibit ECs proliferation and ReEndo [1, 23, 24]. Therefore, control of post-injury inflammatory response represents a desirable approach to control restenosis. IL-10 was originally described as an anti-inflammatory cytokine [25, 26]. IL-10 global knock out mice exhibit unregulated inflammatory activity, which is exemplified by enhanced TNF accumulation and thus is associated with a variety of pathogenic outcomes including atherosclerosis [27–29]. At molecular level IL-10 suppresses mRNA expression of inflammatory cytokines mainly via post-transcriptional mRNA destabilization. We have recently shown that IL-10 inhibited TNF mRNA stabilizing protein HuR both in vitro and in vivo [13, 29]. The IL-10 regulation of HuR might be occurring through various signaling pathways. Previously we have shown that IL-10 inhibits a panel of pro-inflammatory cytokines through suppression of P38 MAPK [12, 13, 29]. Evidence regarding the anti-inflammatory effects of IL-10 to the neo-intimal thickening and restenosis following arterial injury is limited with some studies showing that IL-10 plays an important role in the suppression of intimal hyperplasia and restenosis and atherosclerosis [13, 29, 30]. Furthermore, it has also been shown that IL-10 treatment strongly inhibits macrophage infiltration, proliferation and reduced intimal growth after balloon angioplasty in hypercholesterolemic rabbits [31]. None of these studies, however, looked the effect of IL-10 on endothelial cells proliferation, survival and function after arterial injury. In this study, IL-10 KO mice exhibited reduced ReEndo and enhanced both medial and intimal area after coronary denudation. Furthermore, our in vivo data suggest the excessive proliferation and recruitments of vascular smooth muscle and inflammatory cells at site of injury. In the same line others and we have previously shown that IL-10 deletion strongly induced mobilization of inflammatory and smooth muscle cells at injury site, which secretes pro-inflammatory cytokines, like TNF [13, 29, 30]. As inflammation play an important role in this pathology, IL-10 can reduce the recruitment and proliferation of inflammatory cells and VSMCs. Interestingly; our data clearly suggests that IL-10 treatment significantly reduced neo-intimal hyperplasia as well as vascular smooth cell proliferation. The major limitation of drug-associated stents is their non-selectivity to the target cells. In addition to inhibiting smooth muscle cell proliferation and migration, drugs associated with stent therapy also suppress endothelial cell proliferation and mobility, which may contribute to adverse consequences such as delayed or impaired ReEndo, the need for prolonged dual antiplatelet therapy, and stent thrombosis [32–34]. Thus, despite the clinical benefits of DES in reducing restenosis, there continue to concern about the long-term risk of myocardial infection and cardiac death. Intriguingly, IL-10 strongly activated endothelial cells proliferation. In fact, in addition to its proliferative effects on ECs, it inhibited the proliferation of VSMCs. Our results are distinct from previously published studies as we show new paradigm of IL-10 effects after inflammatory stimulus such as, differential effects on ECs and VSMCs [31]. To the best of our knowledge our studies for the first time show a differential proliferative response of IL-10 on ECs and VSMCs. Inflammatory insults are known to regulate the vascular endothelial cells proliferation by inducing their cell cycle arrest and apoptosis [18, 35, 36]. Our data in this study have shown that IL-10 rescued TNF-induced cell cycle arrest as shown by cells cycle analysis and cyclin-A transcriptional activity. Additionally, IL-10 strongly inhibited TNF-induced endothelial cells death. Our data have shown a very clear indication that IL-10 differential regulates the proliferation of ECs and VSMCs.

In sum, since IL-10 function and signaling are important components for control of inflammatory responses, our data attempts to provide a better mechanism of IL-10 in regulation of endothelial cells function, and may provide insights necessary to develop non-invasive strategies for modulating the vascular repair and other accelerated arteriopathies, including in-stent-induced restenosis.
